# Self-management barriers in recurrent acute pancreatitis: a qualitative study from the health belief model perspective

**DOI:** 10.3389/fpubh.2026.1970484

**Published:** 2026-09-17

**Authors:** Tao Li, Yan Zhou, Lijun Zhou, Xiaoman Wei, Lei Shi, Lanlan Leng, Weili Zhan, Lifeng He, You Yuan, Qiong Wu

**Affiliations:** 1 Department of Emergency, Affiliated Hospital of Zunyi Medical University, Zunyi, Guizhou, China; 2 Department of Critical Care Medicine, Affiliated Hospital of Zunyi Medical University, Zunyi, Guizhou, China; 3 School of Nursing, Zunyi Medical University, Zunyi, Guizhou, China; 4 Nursing Department, Affiliated Hospital of Zunyi Medical University, Zunyi, Guizhou, China

**Keywords:** health belief model (HBM), knowing-doing gap, qualitative study, recurrent acute pancreatitis, self-management barriers

## Abstract

**Objective:**

To explore the self-management barriers and their underlying mechanisms in patients with recurrent acute pancreatitis (RAP) based on the health belief model, and to provide a basis for developing systematic intervention strategies.

**Methods:**

A convenience sampling method was used to select 24 patients with RAP admitted to the Affiliated Hospital of Zunyi Medical University from December 2024 to April 2025. Using the health belief model (HBM) as the theoretical framework, semi-structured interviews were conducted to collect data. The Colaizzi seven-step content analysis method was employed to code, summarize, and extract themes from the interview transcripts.

**Results:**

The mean age of the patients was 47.25 ± 13.74 years. Among them, 14 were male (58.33%) and 10 were female (41.67%). Three core themes and six sub-themes were identified. Theme 1, Perceived Threat, included two sub-themes: perceived susceptibility and perceived severity. Patients generally underestimated the recurrence risk and exhibited a sense of luck, while simultaneously bearing multiple pressures from the consequences of recurrence. Theme 2, Behavioral Evaluation, included perceived benefits and perceived barriers. Although patients recognized the importance of behavior change and formed initial beliefs, they encountered multiple real-world obstacles such as financial burden, work pressure, and social barriers during the translation of knowledge into action, resulting in the core dilemma of the “knowing-doing gap.” Theme 3, Behavioral Enablers, included cues to action and self-efficacy. Patients’ health behaviors were mostly triggered by isolated physical symptom signals without systematic triggering mechanisms. Their self-efficacy showed dynamic fluctuations, which directly affected the sustainability and stability of behavior change.

**Conclusion:**

The self-management dilemmas of patients with recurrent acute pancreatitis run through the three core components of the HBM, manifesting as coexistence of a sense of luck and multiple pressures, the knowing-doing gap, lack of cues, and fluctuating efficacy. It is recommended to design systematic and individualized self-management support programs by strengthening continuous risk perception, reducing behavioral barriers, enhancing self-efficacy, building support systems, and establishing proactive cues to action.

## 1 Introduction

Globally, the incidence of recurrent acute pancreatitis (RAP) is rising each year. This not only increases the burden on healthcare systems but also seriously affects patients’ physical and mental health (1–4). Studies show that the recurrence rate of RAP ranges from 20 to 40% (5–7). Some patients experience a second episode within 1 year after the first attack (8), and the more recurrences they have, the higher the risk of progressing to chronic pancreatitis (9, 10). Triggers for recurrence include dietary indiscretion, inadequate treatment of biliary tract diseases (11, 12), hypertriglyceridemia (13–15), and other pathological factors, as well as insufficient disease management knowledge and poor self-management adherence (16–18).

Clinical practice shows that patients’ self-management behaviors are key to controlling RAP recurrence. However, most patients currently face the problem of “knowing but not doing, and doing but not persisting”. That is, they know what they should do but cannot put it into action, or they take action but cannot stick with it. This problem has long existed and has become a difficulty in clinical nursing and chronic disease management (19–21). The key to solving this problem lies in whether patients can actively recognize the severity and susceptibility of disease recurrence, clearly understand the benefits of healthy behaviors, strive to overcome difficulties during implementation to build confidence, and at the same time manage dietary adjustment, regular follow-up, and psychological adaptation well. Only then are patients more likely to transform disease management knowledge into long-term healthy behaviors, thereby reducing the risk of RAP recurrence.

The health belief model (HBM) is a classic theoretical framework for analyzing health-related behavioral decisions. The model explains the formation process of individual health behaviors through six dimensions: perceived severity, perceived susceptibility, perceived benefits, perceived barriers, self-efficacy, and cues to action. It provides theoretical support for research on chronic disease self-management (22). Previous studies have confirmed that the HBM has obvious effects in guiding behavioral interventions for patients with chronic diseases such as diabetes and hypertension (23–25). However, systematic analyses of self-management in patients with RAP are still relatively rare. Existing studies have primarily focused on acute phase treatment and dietary guidance (20), while research on the psychological and behavioral mechanisms of long-term self-management in RAP patients, HBM-based risk perception and behavioral appraisal, as well as daily cues to action, remains very limited.

Therefore, this study uses the HBM as its theoretical framework and adopts qualitative methods to analyze the cognitive characteristics and behavioral status of RAP patients in recurrence control. It focuses on the main perceptual contradictions, self-efficacy deficiencies, and limitations in cues to action, aiming to reveal the mechanism of the “cognition to behavior” rupture. The study provides a basis for developing systematic and individualized self-management support programs, facilitating the translation of knowledge into behavior, and ultimately improving self-management abilities, reducing recurrence rates, and enhancing long-term prognosis for RAP patients.

## 2 Participants and methods

### 2.1 Research methods

This single-center, qualitative descriptive study adhered to the Consolidated Criteria for Reporting Qualitative Research COREQ guidelines for qualitative research reporting in its data analysis and result reporting (26, 27).

### 2.2 Study participants

Convenience sampling was used to select patients with RAP who had been admitted to the ICU during prior episodes and treated at a university-affiliated hospital. The study was conducted from December 2024 to April 2025. During this period, eligible participants were recruited through two approaches: direct recruitment during hospitalization and recruitment through outpatient follow-up visits. The inclusion criteria were as follows: (1) age > 18 years, diagnosed with RAP with at least one documented recurrence, and being in a stable condition; (2) good verbal expression and comprehension abilities; (3) voluntary participation with signed informed consent. Recurrence was defined according to published consensus criteria: (1) interval between attacks >3 months; (2) complete or near-complete resolution of the first episode; and (3) no evidence of underlying chronic pancreatitis (28). Episodes during the same hospitalization were excluded. The exclusion criteria were as follows: (1) mental disorders, cognitive impairment, or inability to participate in the interview due to medical reasons; (2) severe hearing or speech impairments that prevented effective communication; (3) concurrent severe heart, lung, liver, kidney, or other organ failure; (4) concurrent participation in other interventional clinical studies or explicit refusal to participate. Two interview formats were used: face-to-face interviews and online telephone conference interviews, with the choice of format determined based on the participants’ physical condition and personal preference.

### 2.3 Theoretical framework

This study employed health belief model (HBM), developed by social psychologists Hochbaum, Rosenstock, and Kegels, as its theoretical framework to systematically investigate self-management barriers in patients with RAP from the HBM perspective (29, 30), as illustrated in Figure 1. The model integrates individual health behavior decision-making into three core components: Perception, corresponding to HBM’s Perceived Threat; Evaluation, corresponding to HBM’s Behavioral Appraisal; and Facilitation, corresponding to HBM’s Behavioral Enablers. Six specific dimensions are encompassed: (1) perceived susceptibility, manifested as patients’ underestimation of recurrence likelihood with a sense of luck; (2) perceived severity, reflected in patients’ fear of recurrence consequences and the multiple pressures they bear; (3) perceived benefits, referring to patients’ recognition of the significance of behavioral change and formation of initial beliefs; (4) perceived barriers, representing the multiple real-world obstacles patients encounter when translating knowledge into action, constituting the core dilemma of the “knowing-doing gap”; (5) cues to action, characterized by patients’ reliance on isolated physical symptom signals without systematic triggers; in this study, social support and technology platforms are understood as modifying or enabling factors; and (6) self-efficacy, presenting fluctuating confidence that determines the sustainability and stability of their behavioral change. Based on this theoretical framework, this study systematically constructed interview guidelines across the six dimensions, aiming to reveal the mechanism underlying the “cognition-to-behavior” rupture and to deeply explore the cognitive characteristics and behavioral mechanisms of self-management barriers in patients with RAP.

**Figure 1:**
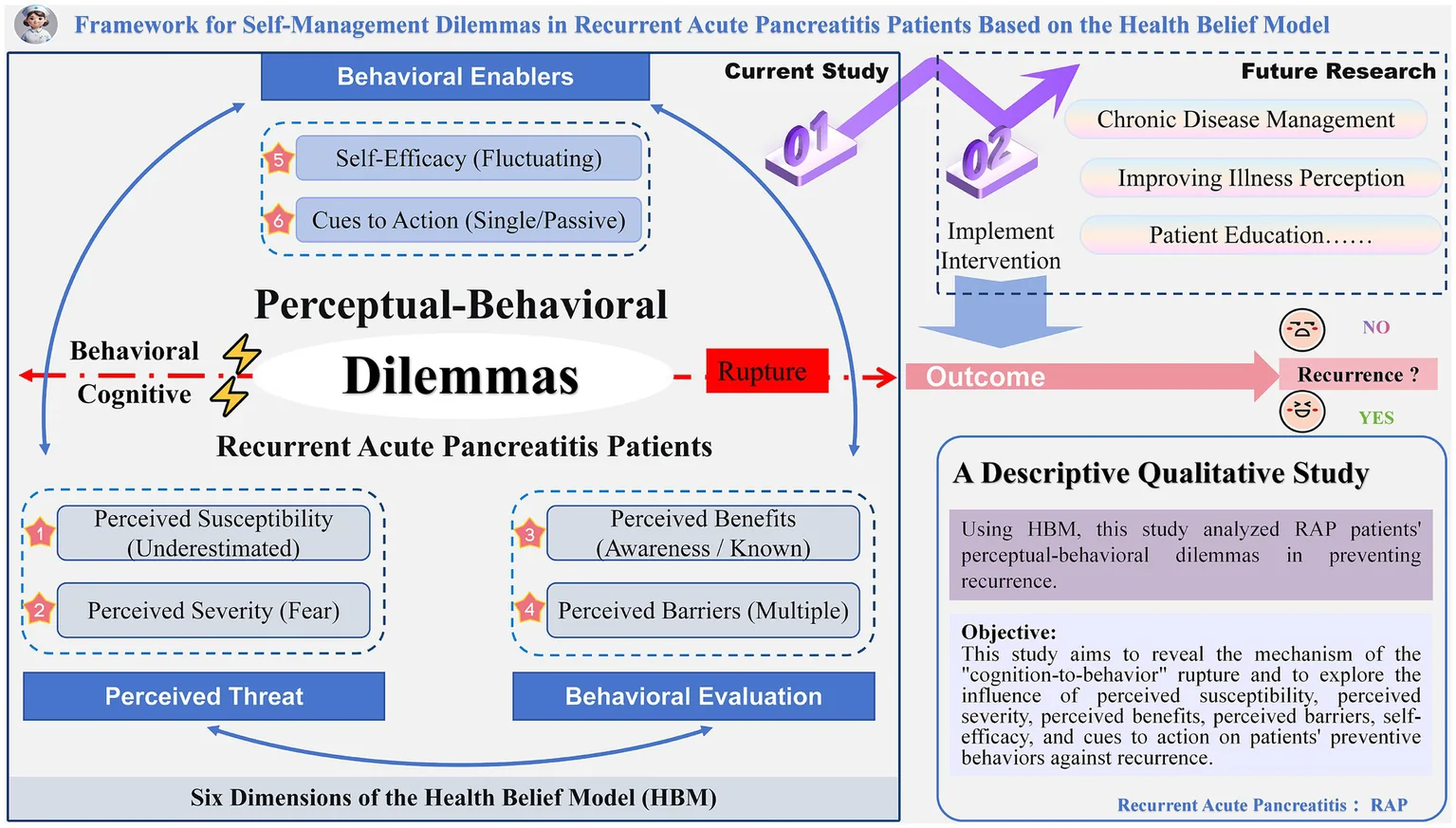
Framework for self-management dilemmas in recurrent pancreatitis patients based on the health belief model.

### 2.4 Research instruments

The instruments used in this study included a semi-structured interview guide, an AI-powered smart voice recorder, a backup mobile phone, a laptop computer, paper and pen, NVivo software, and Excel and SPSS statistical software. The AI-powered smart voice recorder was equipped with dialect recognition and real-time transcription functions. The laptop computer, paper, and pen were used to document interview data and key points. NVivo software assisted in coding and thematic extraction of interview transcripts, while Excel and SPSS software were used for descriptive statistical analysis of the sample data.

### 2.5 Interview outline development

The research team initially developed six semi-structured interview questions based on the six core dimensions of the HBM. After conducting pre-interviews and team discussions, one comprehensive open-ended question was added, resulting in the final interview outline, as shown in Table 1.

**Table 1:** Interview outline based on the health belief model theoretical framework.

Interview question	Dimension	Purpose
1. In your opinion, what specific impacts does recurrent pancreatitis have on your life, work, psychology, and family?	Perceived severity	To assess patients’ fear of recurrence consequences and the multiple pressures they bear
2. What behavioral habits or physical changes do you think may lead to your recurrence? Were there any warning signs before recurrence?	Perceived susceptibility	To identify patients’ underestimation of recurrence risk with a sense of luck and specific triggers
3. What measures have you taken to prevent recurrence, and how were these measures formed?	Perceived benefits	To understand patients’ recognition of the benefits of healthy behaviors and the formation of initial beliefs
4. What are the biggest challenges or difficulties you encounter in preventing recurrence (e.g., dietary control, work pressure, social barriers, financial burden)?	Perceived barriers	To reveal the real-world obstacles patients face when translating knowledge into action
5. What specific help do you hope to receive from healthcare providers, family members, or technical platforms to prevent recurrence?	Cues to action	To clarify the triggers patients rely on and the systematic support they lack
6. How confident are you in your ability to effectively control pancreatitis recurrence in the future? What factors influence your confidence?	Self-efficacy	To explore the characteristics of fluctuating confidence and its impact on behavioral sustainability
7. In addition to the above, are there any other barriers, unique experiences, or reflections you would like to share?	Open-ended	To encourage patients to share additional barriers, unique experiences, and reflections in disease management

### 2.6 Implementation and data collection

Data collection was completed by four systematically trained researchers. Before the interview, the researchers explained the purpose, procedures, participants’ rights, privacy protection measures, and the principle of voluntary participation to the patients and their families in detail, ensuring that they fully understood and agreed to participate. Written informed consent was obtained for face-to-face interviews, while verbal informed consent was obtained for online interviews. Participants could withdraw from the study at any time.

The interview format, either face-to-face or online, was selected based on the patients’ physical condition and personal preference. Face-to-face interviews were conducted in a quiet meeting room or ward area, while online interviews were conducted via telephone, WeChat voice call, or Tencent Meeting. Previous research has confirmed the reliability and feasibility of online video interviews for qualitative data collection (31, 32). Each interview lasted 10 to 50 min and was conducted in Chinese. The entire interview process was recorded using an AI powered smart voice recorder and a backup mobile phone.

Within 24 h after recording, the interviews were transcribed verbatim and organized to ensure data completeness and accuracy. All interview data were kept strictly confidential and anonymized. Participant IDs were represented by the letter P followed by a number, such as P1, P2, P3, up to Px. Personal information and interview content were used solely for academic research and will not be disclosed.

### 2.7 Data analysis

This study employed the Colaizzi seven-step content analysis method in combination with the HBM theoretical framework to systematically process, code, summarize, and extract themes from the interview transcripts. The specific steps are shown in Figure 2.

**Figure 2:**
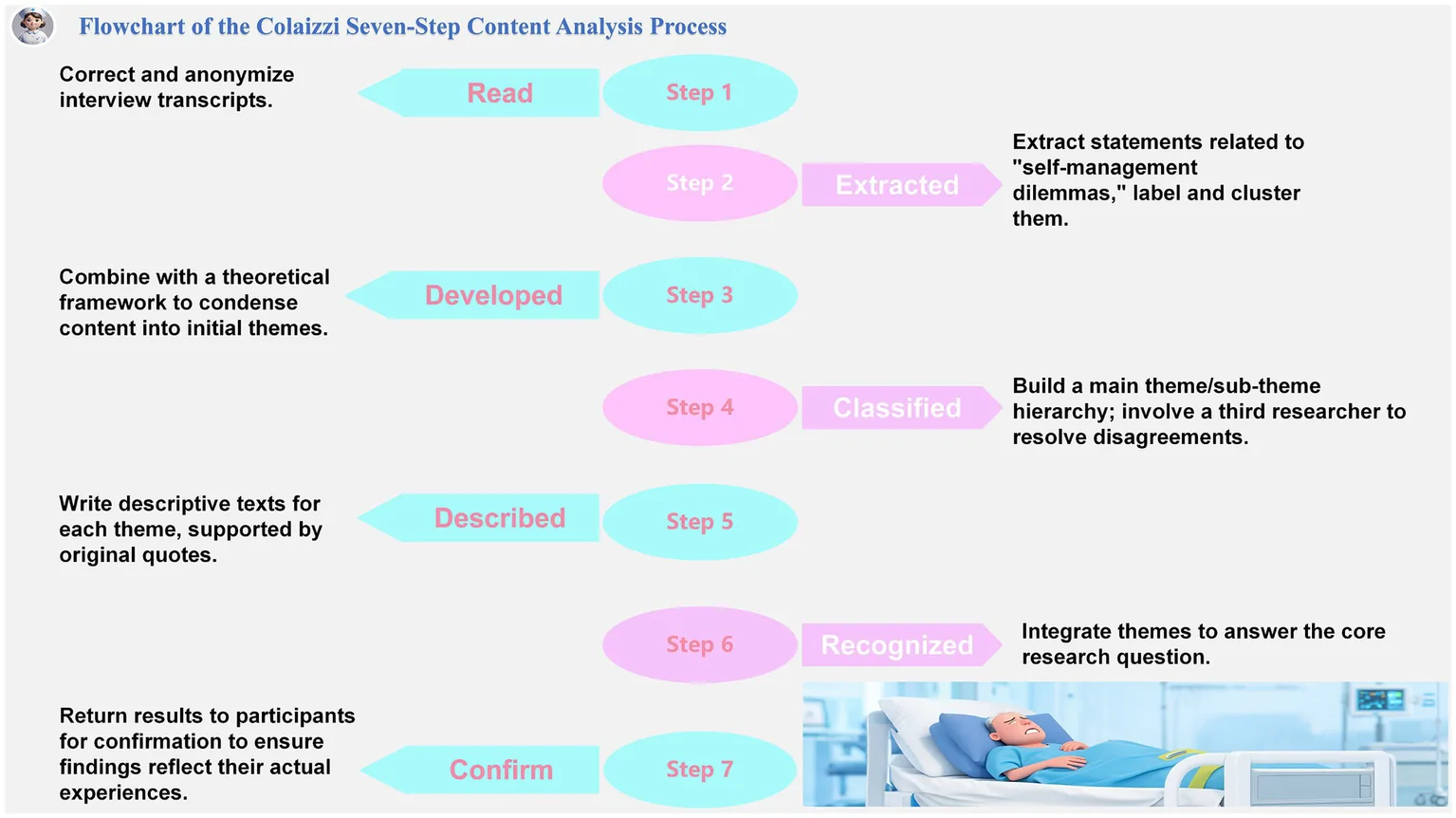
Flowchart of the Colaizzi seven-step content analysis process.

#### 2.7.1 Step 1: Correction and anonymization

Within 24 h after the interview, the researchers listened to the original recordings, checked and corrected errors in the automatically transcribed drafts, produced clean transcripts, and performed anonymization.

#### 2.7.2 Step 2: Extraction of significant statements

Two researchers cross-read the clean transcripts, identifying original segments directly related to “self-management dilemmas in patients with recurrent pancreatitis.” They assigned initial labels, clustered similar content, and extracted meaningful units.

#### 2.7.3 Step 3: Formation of meaning units

The research team repeatedly reviewed the transcripts, extracting content related to the research questions through annotation and coding. The meaning units were mapped to the core characteristics of the patients based on the six dimensions of the health belief model: perceived susceptibility, perceived severity, perceived benefits, perceived barriers, cues to action, and self-efficacy.

#### 2.7.4 Step 4: Clustering and preliminary themes

Similar meaning units were grouped together to construct a hierarchical framework of main and sub-themes. In the event of disagreements, a third researcher was consulted to discuss and reach a consensus. Three major themes were preliminarily identified: perceived threat, behavioral appraisal, and behavioral enablers.

#### 2.7.5 Step 5: Comprehensive theme description

Descriptive text was written for each theme, with key original statements embedded as supporting evidence. Specifically, perceived threat included perceived susceptibility (underestimation of recurrence risk and a sense of luck) and perceived severity (fear of recurrence consequences and multiple pressures); behavioral appraisal included perceived benefits (recognition of the significance of behavior change) and perceived barriers (real-world obstacles and the knowledge-action gap); behavioral enablers included cues to action (reliance on isolated signals and lack of systematic triggers) and self-efficacy (fluctuating confidence and determination of behavioral sustainability).

#### 2.7.6 Step 6: Extraction of essential structure

All themes were integrated to summarize and reveal the core essence of the phenomenon, which was identified as a structural rupture between cognition and behavior, manifested as underestimation of recurrence risk, multiple real-world obstacles, fluctuating self-efficacy, and lack of systematic cues to action.

#### 2.7.7 Step 7: Participant validation

The final theoretical framework and representative quotes were returned to the participants for confirmation. Feedback was solicited to ensure that the research findings faithfully and objectively reflected the patients’ actual experiences. Following this, the data analysis was finalized.

### 2.8 Researcher characteristics, reflexivity, and quality control

The interviewers were ICU nurses from the Pancreatitis Diagnosis and Treatment Center in Guizhou, directly caring for critically ill acute pancreatitis patients. They maintained reflexive logs to reduce bias, discussing assumptions with the team. A maximum variation strategy was used to select patients with diverse backgrounds. Pre-interviews were conducted with at least five patients to adjust the interview outline. Interviews were audio-recorded in a quiet setting, with family members allowed to accompany but not contribute to the analysis. Transcripts were returned to patients within 24 h for verification. After independent coding by two researchers, themes were cross-checked and extracted using the HBM. Regular reflective discussions ensured authenticity and trustworthiness.

## 3 Results

A total of 32 RAP patients were invited. Three were excluded due to time and location constraints; two refused for privacy reasons; two had interviews shorter than 10 min; and one did not complete the interview. The final sample included 24 patients, with a mean age of 47.25 ± 13.74 years. Interviews lasted 11 to 50 min, with a mean duration of 30.29 ± 10.77 min; 14 were face-to-face and 10 were online; no substantial differences in thematic coverage were observed between the two modalities. The sample size was justified based on Morse’s guidelines (33), which recommend 20–30 participants for qualitative exploratory studies, and is consistent with similar studies (34, 35). Thematic saturation was reached after 20 interviews, with the remaining four interviews confirming existing themes. Among them, 14 were male (58.33%), and 10 were female (41.67%). Fourteen lived in urban areas (58.33%) and ten in rural areas (41.67%). Education levels: 10 had associate degrees or higher, 7 had junior/high school, and 7 had primary school. Insurance types: 15 had Urban and Rural Resident Medical Insurance, 8 had Employee Medical Insurance, and 1 had self-paid. Recurrence: 11 had their first recurrence, 10 had their second, and 3 had ≥3 recurrences. Smoking and drinking history: 8 (33.33%) each. Etiology: 12 biliary, 7 hyperlipidemic, 1 alcoholic, and 4 mixed, as shown in Table 2.

**Table 2:** Demographic and clinical characteristics of participants (*N* = 24).

ID	Gender	Age	Education	Occupation	Insurance*	Residence	Recurrence	Smoking	Drinking	Etiology
P1	Male	46	High school	Truck driver	URMI	Urban	Second	No	No	Biliary
P2	Male	49	Bachelor’s degree	Civil servant	EMI	Urban	≥3 times	No	No	Biliary
P3	Female	36	Junior high school	Restaurant owner	URMI	Rural	First	No	No	Hyperlipidemic
P4	Male	48	Junior high school	Courier	URMI	Urban	Second	Yes	Yes	Alcoholic, Hyperlipidemic
P5	Female	76	Primary	Unemployed	URMI	Rural	Second	No	No	Biliary
P6	Female	73	Primary	Unemployed	URMI	Rural	First	No	No	Biliary
P7	Female	52	Junior high school	Self-employed	URMI	Urban	Second	No	No	Hyperlipidemic
P8	Male	55	Primary	Small shop owner	URMI	Urban	First	No	No	Biliary
P9	Male	48	High school	Self-employed	URMI	Rural	First	No	Yes	Alcoholic, hyperlipidemic
P10	Male	39	Bachelor’s degree	Civil servant	EMI	Urban	Second	Yes	Yes	Biliary
P11	Male	46	Bachelor’s degree	Teacher	EMI	Urban	First	Yes	No	Biliary, hyperlipidemic
P12	Female	51	Primary	Unemployed	URMI	Rural	≥3 times	No	No	Biliary
P13	Male	50	Junior high school	Security guard	URMI	Rural	Second	Yes	Yes	Hyperlipidemic
P14	Female	28	Bachelor’s degree	Civil servant	EMI	Urban	First	No	No	Biliary
P15	Male	36	Bachelor’s degree	Programmer	EMI	Urban	First	Yes	No	Biliary
P16	Male	51	Primary	Security guard	URMI	Rural	First	No	Yes	Alcoholic
P17	Female	27	Associate degree	Teacher	EMI	Urban	First	No	No	Hyperlipidemic
P18	Male	38	Associate degree	Delivery rider	URMI	Urban	Second	Yes	No	Biliary, hyperlipidemic
P19	Female	68	Primary	Unemployed	URMI	Rural	First	No	No	Biliary
P20	Male	27	Associate degree	Freelancer	Self-paid	Urban	Second	Yes	Yes	Hyperlipidemic
P21	Male	26	Bachelor’s degree	State-owned employee	EMI	Urban	Second	Yes	Yes	Hyperlipidemic
P22	Female	55	Primary	Farmer	URMI	Rural	≥3 times	No	No	Biliary
P23	Female	61	Junior high school	Farmer	URMI	Rural	Second	No	No	Biliary
P24	Male	48	Bachelor’s degree	Civil servant	EMI	Urban	First	No	Yes	Hyperlipidemic
* URMI, urban and rural resident medical insurance; EMI, employee medical insurance; self-paid = no medical insurance, all expenses paid by the patient.										

Based on the HBM, this study systematically summarized and integrated the interview data, identifying three core themes and six sub-themes, as shown in Figure 3. The perceived threat dimension reflects patients’ tendency to underestimate and fear the risk of recurrence, mainly influenced by both perceived susceptibility and perceived severity. In the behavioral evaluation dimension, patients struggled between the benefits of behavior change and the barriers they face, particularly the conflict between recognizing the importance of change and dealing with real-world obstacles. The behavioral facilitation dimension reveals the lack of support and fluctuating self-efficacy, which affects the stability of behavior continuity. Together, these themes form the complete process of self-management, with “perception, evaluation, facilitation” forming a continuous behavioral path.

**Figure 3:**
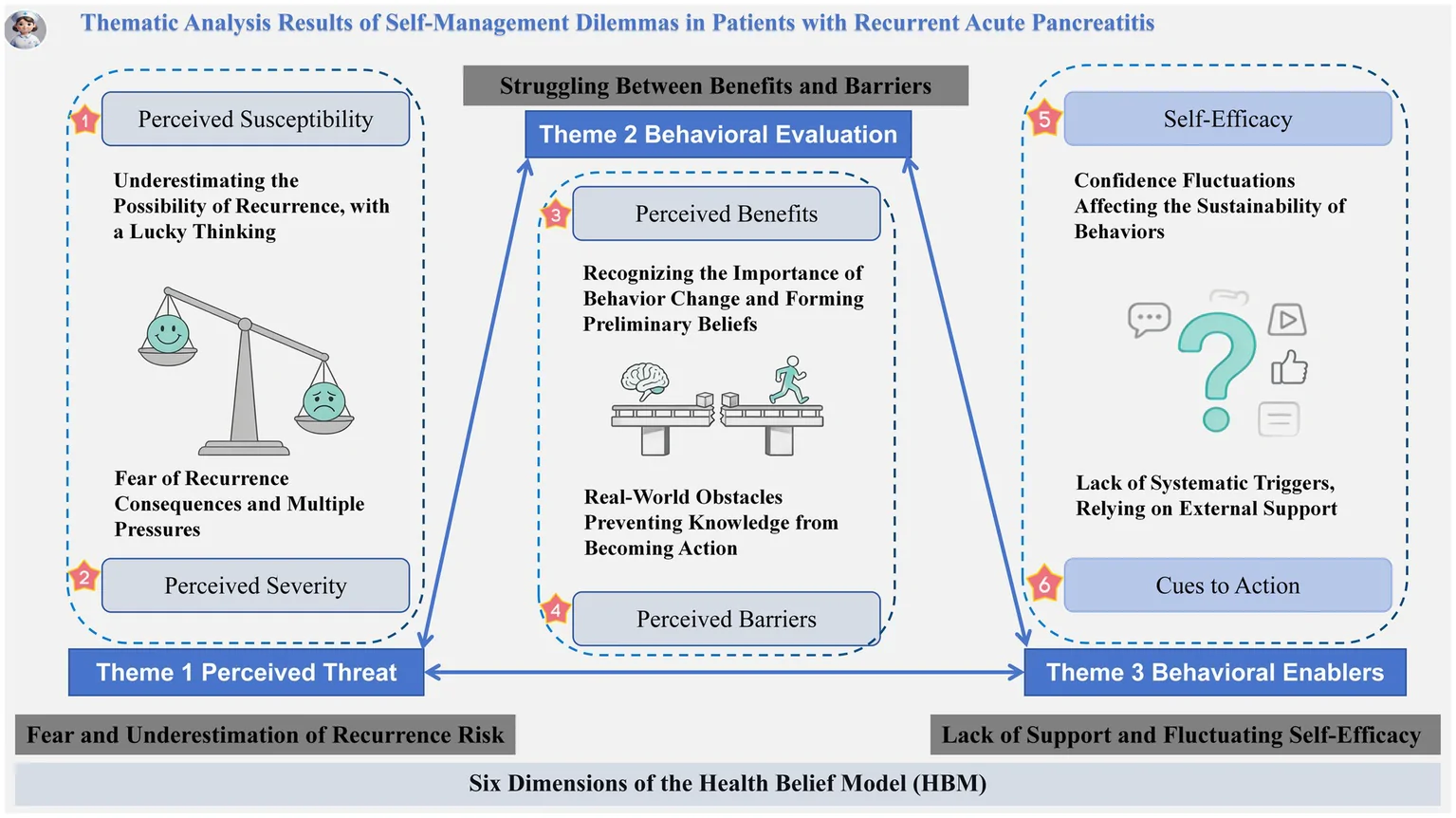
Thematic analysis results of self-management dilemmas in patients with recurrent acute pancreatitis.

### 3.1 Theme 1 perceived threat: fear and underestimation of recurrence risk

The interview results showed that patients’ awareness of the risk of pancreatitis recurrence included two aspects: perceived susceptibility and perceived severity. While patients generally recognized the risk of recurrence, their awareness tended to underestimate the actual risk. At the same time, they expressed varying degrees of concern and stress about the consequences of recurrence.

#### 3.1.1 Perceived susceptibility: underestimating the possibility of recurrence, with a lucky thinking

Most patients (18/24) acknowledged that they were at risk of recurrence, but their awareness of this risk was unstable, manifested in fragmented awareness of triggers and the coexistence of lucky thinking.

P8: "When I was hospitalized, I listened to everything the doctor said. After being discharged, I followed the advice for a while, but eventually I felt fine and thought I was okay. I ate and drank as usual, and then I was hospitalized again."

P14: "I thought I was taking good care of myself, eating well, not drinking or smoking. I thought I wouldn’t relapse. But unexpectedly, I relapsed. I can’t understand why."

The majority of patients (11/24) attributed the recurrence to improper diet or drinking.

P13: "After my blood fat went back to normal, I returned to my usual eating habits. I ate greasy food every day and didn’t have regular check-ups."

P16: "I’m someone who can’t do without alcohol. Working the night shift as a security guard often messed up my sleep, so I drank some wine to help me sleep. I thought it wouldn’t be a problem if I drank a little, but I didn’t expect to relapse."

P24: "Two days before the relapse, I drank white wine, beer, and spicy grilled food. The day before, I ate particularly spicy food."

Some patients thought that changes in physical condition or early signs of recurrence were important triggers.

P3: "I’m usually very careful with my diet and eat lightly, but I noticed that I got sick after I caught a cold. When the body’s resistance is low, it’s easy for the disease to occur."

P2: "I could feel that I was about to relapse. I had a slight pain and discomfort in my stomach."

A few patients mentioned irregular medication use and lack of follow-up as triggers.

P7: "I thought I had fully recovered, so I didn’t need to take medicine regularly. I missed a few days in a row and didn’t return to the hospital for check-ups. As a result, I suddenly became ill, and my condition gradually worsened."

Some patients showed a lack of understanding of the disease and exhibited a significantly low risk perception.

P20: "I thought my health was great, so I didn’t even join the medical insurance, but unexpectedly, I relapsed twice."

P13: "I had stopped smoking for a while, but then I began smoking a bit every now and then."

P10: "Actually, I felt a little discomfort in my stomach a few days before the relapse, but I didn’t take it seriously. I thought it would pass. If I had known it would get this serious, I would have gone to the hospital earlier."

The results showed that although patients were aware of the possibility of recurrence, they still underestimated the risk and exhibited cognitive biases in their behavioral decisions.

#### 3.1.2 Perceived severity: fear of recurrence consequences and multiple pressures

Most patients reported a high perception of the severity of the disease’s consequences. This impact was felt in various aspects, including health, psychological, social, economic, family, and future expectations.

Regarding health and quality of life, patients generally believed that the disease significantly affected their quality of life.

P1: "I used to drive trucks for over ten hours a day with no problem, but now I’m afraid to drive because I’m afraid of having an attack."

P2: "It affects both my work and life a lot. I have to be especially careful with my diet, and I can’t stay up late. It feels very troublesome."

P8: "Now I’m afraid to eat and drink. Life has lost all enjoyment. I used to think life was all about eating and drinking, but now even those things have become a burden."

P10: "Today, when I eat just a little, my stomach bloats. I’m starting to wonder if I’m relapsing."

Psychologically, some patients showed continuous anxiety and uncertainty.

P12: "Sometimes, I don’t feel anything when standing or sitting, but when I press on my upper left abdomen, it hurts. I don’t know when I’ll relapse, which causes a lot of mental pressure and fear."

P14 also added, "I’m afraid of relapsing, and I can’t sleep at night, wondering if it will turn into cancer."

P22: "Whenever my stomach hurts a little, I get nervous, afraid that I’m about to relapse."

P7: "This disease is like a time bomb. You don’t know when it will explode. I’m always on edge."

P11: "Before each check-up, I feel very nervous, fearing bad results, fearing the doctor will say I need to be hospitalized."

Socially, some patients experienced social isolation due to dietary restrictions.

P4: "I used to drink with friends, talking about everything. But now I can’t drink, so I almost never eat with them anymore."

P9: "I don’t dare to go to friends' gatherings. When I go, I watch others eat and drink, while I can only drink water. Gradually, they stopped inviting me."

"Also, this disease has affected my social life. I can’t drink anymore, and it’s damaging my business. Without alcohol, my friends aren’t as close as before."*

Economically, some patients faced heavy financial burdens.

P11: "I’m the main economic provider at home. Since I got sick, I’ve had great difficulties at work, which has worsened our family’s financial situation."

P22: "I’ve had many relapses, and with two kids and a wedding to plan, the financial pressure is huge."

P16: "Every time I’m hospitalized, it costs a lot of money. After insurance, I still have to pay a large amount out of pocket. Now my family is financially tight."

P8: "The ICU hospitalization costs tens of thousands, and plasma exchange is very expensive. It feels like a wasted year."

P19: "I’m 68 years old with no income, and I need to pay for medicine and check-ups. I don’t want to burden my children."

Family relationships also influenced patients’ perceptions of support.

P21: "I’m not too concerned about my diet anymore, and sometimes my family isn’t as concerned as when I was first discharged. I worry that I might relapse."

P6: "At first, my family was very nervous. After a while, they became annoyed. Sometimes I can’t control myself and want to eat something, but they don’t care anymore."

P5: "My spouse eats vegetarian food with me, and it hurts me to see them losing weight."

Regarding future expectations, some patients felt confused and hopeless.

P2: "I don’t know when this disease will end. It feels like I’ll live like this for the rest of my life."

P14: "Will I ever fully recover? What will happen if I don’t do anything?"

P20: "I’m still so young, and my future is long. I really don’t know what to do."

P21: "The doctor said it can be controlled and won’t relapse, but I still feel uncertain."

The interview results indicated that patients had a strong perception of the disease’s consequences, but this perception was often expressed as multidimensional, ongoing pressure and burden that deeply affected their daily functioning, social roles, and future expectations.

### 3.2 Theme 2 behavioral evaluation: struggling between benefits and barriers

In self-management, patients’ cognitive assessments of health behaviors can be summarized as perceived benefits and perceived barriers. While patients generally recognized the value of behavior change, multiple barriers in practice hindered the translation of knowledge into action. The imbalance between benefits and barriers constitutes the essence of the “knowledge-action gap” in self-management.

#### 3.2.1 Perceived benefits: recognizing the importance of behavior change and forming preliminary beliefs

Patients generally recognized the importance of healthy behaviors in preventing recurrence, especially dietary changes. The majority viewed dietary adjustments as the primary means to prevent recurrence, reflecting a shift from passive acceptance to active change.

Some patients changed their dietary habits through psychological adjustment.

P18: "To live longer, I changed my eating habits. I turned my dislike into a preference. Before I got sick, I liked greasy food, but now I can’t even look at greasy food without feeling disgusted."

P23: "I don’t feel much pressure now. My kids are married, so I just focus on resting and regular check-ups."

P17: "My colleagues say I look much better after losing weight, and I feel lighter physically."

Some patients emphasized the importance of eating lightly, eating small meals more frequently, and exercising.

P21: "Eat light, and have smaller, more frequent meals."

P24: "Don’t overeat, and exercise more."

P17: "Combine seafood with soy sauce, eat more chicken, fish, shrimp, tofu, cucumbers, bananas, and lettuce to make juices."

Additionally, some patients realized that, besides dietary control, lifestyle and emotional states also played a crucial role in preventing recurrence.

P20: "Mainly, I should get my routine right, avoid alcohol and smoking, rest well, control emotions, and avoid spicy or greasy food."

P2: "We should learn to cherish our own health. It’s not just about us but also about our family."

Some patients recognized the importance of external supervision and self-monitoring tools.

P10: "I held a family meeting at home, and my family supervises my habits. I have a lot of confidence in this change."

P24: "I think it’s essential to control my eating habits, and my family should help and supervise me."

P15: "I’m a programmer, and I’m used to solving problems with technology. I hope there could be a health management platform to supervise and remind me, like the 'Pancreatic Check, ' although some functions require payment."

Some patients reflected on their past unhealthy lifestyle and changed their behavior after experiencing the pain of the disease.

P9: "If I had known how painful this disease would be, I wouldn’t have drunk so much. I will eat less in the future."

P20: "I used to love eating meat, eating quickly, drinking soda, and having an irregular lifestyle. Now that I think about it, my lifestyle was unhealthy, and that led to this disease. I need to change my lifestyle."

In general, patients have developed a preliminary belief in health, recognizing the value of healthy behaviors, but this recognition remains largely at the cognitive level and has not yet translated into stable behavioral practice.

#### 3.2.2 Perceived barriers: real-world obstacles preventing knowledge from becoming action

Patients face multiple real-world obstacles when trying to convert their health awareness into actual behavior. These obstacles include financial burdens, work pressure, social barriers, psychological struggles, and dietary restrictions.

The most common barrier is the economic burden, as many patients believe medical costs are a major obstacle, especially for those from low-income families who are concerned about the high costs.

P16: "I’m the main economic provider in my family. Since I got sick, it’s been very difficult at work, which has increased the financial burden on my family."

Patients also mentioned that busy work and family responsibilities limited their time for healthy behaviors.

P11: "I know what I should do as a pancreatitis patient, but as a high school teacher and head of class, I’m so busy that I don’t have time, and sometimes I just can’t follow the doctor’s orders."

P2: "I don’t have time because of work pressure. I don’t get enough rest at noon, and I have meetings until late at night. My meals are very irregular."

P18: "I’m a delivery driver, and I’m always on the road. I don’t have time to eat, and sometimes I just grab a buffet, which is oily and salty. I know it’s wrong, but I can’t bear not eating."

P13: "I’m a security guard. My shifts are long and irregular, and after a night shift, I just want to eat something hot. I don’t have time to eat light food."

Patients also felt social difficulties, fearing they could not fit in socially due to the disease.

P9: "This disease means I can’t drink, but my job is at a winery, and both my social and business life have been greatly affected."

P18: "There was a time when my nasal feeding tube was very uncomfortable, and my throat felt like something was stuck. I couldn’t swallow or spit it out, and every day my nose and throat bled. I couldn’t go out at all."

Some patients said that psychological struggles and emotional fluctuations affected their desire for food and self-control.

P14: "When I see delicious food, I really want to eat it, but I can’t. I don’t know if I can control myself in the future."

P16: "I can’t control some of my habits. I’ve liked drinking white wine for decades, and sometimes I can’t help myself."

P6: "I’m 73 years old. At first, my family was very concerned, but over time, they became annoyed. Sometimes, I want to eat something, but they don’t care anymore. They probably think I’m so old that I shouldn’t deprive myself too much."

The trauma experience in the ICU also left deep psychological scars on patients.

P18: "I stayed in the ICU for more than ten days with tubes all over my body. The fear of it will never be forgotten. When I was discharged, I swore I would never eat recklessly again. But after a long time, I sometimes can’t control myself when I crave food."

Some patients believe that health issues led to dietary restrictions, which changed their original eating habits.

P17: "Before, I could enjoy food freely. Now, I have this disease and always get diarrhea. I can’t eat many of the foods I used to enjoy."

Patients face many obstacles: economic, work-related, social, psychological, and family-related, which prevent them from consistently translating their health awareness into actions, leading to a “knowledge-action gap.” Further exploration of the factors facilitating their behavior reveals that patients’ behavioral cues and self-efficacy are also significantly lacking, with support and efficacy being key elements in maintaining behavior change. These factors form the third theme of this study.

### 3.3 Theme 3 behavioral enablers: fluctuating self-efficacy and lack of support

Patients’ health behaviors are often triggered by isolated bodily symptoms and lack a systematic mechanism. At the same time, their self-efficacy fluctuates dynamically. The insufficient support systems in both areas directly affect the continuity and stability of behavioral change.

#### 3.3.1 Self-efficacy: confidence fluctuations affecting the sustainability of behaviors

Self-efficacy is a key factor in whether patients can transform their health awareness into sustained actions in the face of recurrence risk. This study found that patients’ self-efficacy is not stable; it fluctuates dynamically, directly influencing the sustainability of their behaviors, such as dietary control, regular medication, and exercise.

Patients with high self-efficacy often gradually developed a sense of control over disease management through positive feedback from their behavioral practices.

P8: "I’ve been eating lightly for half a year, and my stomach hasn’t hurt again. Now I feel like I can manage my health better. I’m more confident."

P2: "Every time my check-up results are good, I trust more. As long as I maintain my diet and routine, pancreatitis won’t bother me."

Some patients’ self-efficacy built up dynamically, initially struggling to change behaviors but gradually developing self-control by adjusting strategies and self-talk.

P9: "At first, I felt that I couldn’t control my cravings, but later, I found dishes that were both light and tasty. I realized I could control myself."

P18: "Before, I couldn’t resist the smell of oil, but now when I see it, I immediately think, 'This will cause me pain, ' and I’ve gradually built this self-control."

A few patients experienced a complete shift in their overall attitude toward life, further strengthening their self-efficacy stability.

P24: "After being discharged, I controlled my diet and successfully lost weight … My attitude toward life and health has changed, and I cherish life more."

P17: "I lost a lot of weight, and now I feel better than before, both physically and mentally."

In contrast, patients with low self-efficacy showed a lack of control over their future, often falling into passivity and anxiety.

P14: "Will I ever fully recover? What will happen if I don’t do anything?"

P16: "I know I shouldn’t drink, but sometimes I still do."

#### 3.3.2 Cues to action: lack of systematic triggers, relying on external support

Many respondents mentioned that clear guidance from healthcare providers helped control the disease.

P12 and P19 both mentioned: "When I was discharged, they only told me to eat less oil and salt, but didn’t specify what I could or couldn’t eat. I wish they provided some recipes."

P1: "I’m a truck driver. On the road, I mostly eat fast food or instant noodles. Can you tell me specifically what I should eat?"

P6: "One doctor says I can eat a certain food, but the nurse says I can’t. It’s confusing."

Patients were confused about the authenticity of online information.

P21: "Some of the knowledge in the popular science videos on TikTok is wrong."

P4: "Someone said vinegar can lower blood fat, so I tried it, but it caused stomach pain. It’s hard to tell which online information is reliable."

P14: "The dietary tips on Xiaohong Shu vary. Some say I can eat eggs, others say I can’t. I don’t know who to trust."

P11: "I went to a bakery to buy whole wheat bread, but now I’m hesitating. I searched online and found it might contain many additives. I’m not sure if it’s safe to eat."

Patients longed for scientific and actionable dietary advice.

P3: "I have high blood fat, and I hope you can provide a meal plan. I don’t know what I should eat every day."

P11: "Even if there are dietary suggestions, I don’t have time to follow them, and I can’t find suitable food for delivery."

P15: "I’m a programmer, so I’m used to solving problems with technology. I wish there was a health management platform that could supervise and remind me, such as Pancreatic Check, although some features require payment."

Patients also expressed a desire for information about the disease.

P2: "What should I do for prevention? What are the symptoms of recurrence? What should I do immediately if I relapse?"

P18: "I deliver food outside every day and don’t have time to check information. It would be great if the hospital had an app or WeChat public account where I could check anytime."

P12: "A good thing is that I added my doctor on WeChat. If I have questions, I can ask her, but the hospital’s follow-up care doesn’t offer professional disease-related guidance."

Some patients desired peer support.

P7: "Can I be added to a patient communication group? It would be helpful to talk more and supervise each other."

P5: "I’m old. If there were offline support groups, it would be better than figuring it out on my own."

P16: "I’ve been drinking for 30 years, and quitting is hard. It would help if there were patient support to encourage each other."

P2: "The pancreatitis support group taught me a lot of preventive knowledge. I hope more patients will join."

P22: "Sometimes I chat with pancreatitis patients, but these interactions are too scattered. If the hospital could officially organize and correct some misinformation, it would be better."

For these patients, there was a clear gap between their cognition and behavior, which likely increased the risk of recurrence.

## 4 Discussion

### 4.1 Perceived threat and cognitive dissonance: the contradiction between underestimation of recurrence risk and fear of consequences

This study found that RAP patients commonly exhibit a cognitive dissonance characterized by low perceived susceptibility and high perceived severity, which is the cognitive root of their difficulty in adhering to preventive behaviors. According to the HBM, perceived susceptibility is a prerequisite for taking health actions. However, most patients hold a sense of luck and lack awareness of recurrence risk, as exemplified by P16, who believed “a little drink to help sleep should be fine.” Studies have shown that age <60 years, male gender, smoking, and alcohol-related etiology are important risk factors for RAP (36). Chen et al. (19). similarly found that RAP patients commonly experience a sense of uncertainty, with the disease persistently troubling them like an “endless curse.” Meanwhile, patients demonstrate high perceived severity of recurrence consequences, manifested as multidimensional pressures including health, psychological, social, and economic burdens. Lin et al. (37). showed that socioeconomic pressure, physical symptoms, and lower levels of social support are associated with greater fear of disease progression. A meta-analysis reported that the overall prevalence of depression in patients with pancreatic diseases is nearly 40% (38). In this study, P7’s description of the disease as a “time bomb” and P14’s worry about “whether it might turn into cancer” are consistent with these findings. Leventhal et al. (39). pointed out that patients address health threats through four stages: awareness of threat, emotional response, formation of action plans, and adjustment based on feedback. Although patients in this study were aware of the recurrence threat and experienced fear, they misjudged RAP as a one-time acute event rather than a chronic disease, thus failing to form effective action plans and underestimating the long-term value of behavioral interventions. This cognitive dissociation leads patients to fear recurrence without being able to translate that fear into action. Clinical interventions should correct risk perception biases and help patients establish an accurate understanding of the disease timeline.

### 4.2 Behavioral appraisal and the knowing-doing gap: awareness of benefits and barriers hindering preventive behavior

Interviews revealed that RAP patients generally recognize the importance of healthy behaviors in preventing recurrence, particularly in terms of dietary modification. However, Drewnowski et al. pointed out that humans have a natural sensory preference for and psychological dependence on high-fat and high-sugar foods, which places patients in a conflict between rationality and sensibility (40, 41). Chen et al. described the difficult process of patients “fighting against appetite,” while being constrained by objective conditions such as social gatherings, family dietary preferences, and work schedules (19). In this study, P2’s inability to adhere to a healthy diet, P14’s experience of “really wanting to eat but not being able to,” P9’s social and business life being affected by the disease, and P18’s inability to go out due to nasoenteric tube discomfort all confirm this contradiction and reflect the psychological stress caused by social isolation. Chen et al. further pointed out that restrictive dietary requirements often hinder patients’ social participation, especially shared meals, thereby exacerbating social isolation and depressive symptoms (19). Relevant studies have demonstrated that alcohol abstinence can significantly reduce the risk of acute pancreatitis progression (21, 42, 43), but in this study, P16 found it difficult to adhere due to withdrawal reactions and even needed “a little drink to help sleep,” reflecting the resistance of addictive behaviors to health interventions. Meanwhile, some patients achieved positive transformation through profound experiences of disease-related suffering. For example, P18 transformed disgust into preference through psychological adjustment, while P17 and P24 received positive feedback after successful weight loss, completing the shift from “passive restraint” to “active identification.” Boije et al. (44). pointed out that social networks and medical support can effectively promote recovery. In response to the above barriers, clinical interventions should help patients establish short-term achievable behavioral goals, break down long-term benefits into phased outcomes, provide specific and actionable dietary alternatives, assist patients in coping with social pressure, and optimize follow-up procedures to reduce time costs.

### 4.3 Behavioral enablers and sustainability: lack of support and fluctuating self-efficacy constraining behavior maintenance

Interviews revealed that RAP patients have significant deficiencies in both cues to action and self-efficacy, which together constrain the continuity and stability of behavior change. Regarding cues to action, most patients rely solely on physical symptoms such as pain and abdominal bloating as signals for behavior adjustment, indicating a single and passive trigger. In this study, P12 and P19 were only told to “reduce oil and salt” upon discharge without specific dietary guidance; P6 faced confusion from contradictory information between doctors and nurses; P4, P14, P11, and P21 struggled to distinguish the authenticity of online information. Relevant studies have similarly found that patients have difficulty obtaining professional guidance and that the quality of information in patient support groups is inconsistent. Meanwhile, patients expressed a desire for technological platform support, such as P15, and peer support, such as P7, P16, P2, and P22. Chen et al. (19) found that peer support can provide emotional resonance, while Walker and Peterson noted that peer support plays a positive role in chronic disease self-management, though more rigorous research design is needed to validate its effectiveness (45). Regarding self-efficacy, patients exhibited dynamic fluctuations. Patients with high self-efficacy gradually developed a sense of control through positive feedback, as seen in P8, whose stomach never hurt again, and P2, whose follow-up results were consistently good. In contrast, patients with low self-efficacy fell into a vicious cycle of “recurrence-anxiety-relapse-recurrence again,” as seen in P13, who resumed smoking after quitting, and P16, who knew he should not drink but sometimes still did. Conversely, some patients achieved a transformation from passive restraint to active identification, such as P18, who turned disgust into preference, and P24, who cherished life more after successful weight loss. Studies have shown that early discharge of patients with mild acute pancreatitis combined with outpatient services such as remote monitoring or smartphone applications is a safe and feasible measure (46), and the use of mobile health applications can improve health outcomes in patients with chronic diseases by enhancing symptom control (47). In response to the above barriers, clinical interventions can integrate personalized self-monitoring tools such as dietary diaries and medication reminder apps, professionally guided peer support networks, and diversified cue to action systems such as regular follow-ups and family supervision, combined with outpatient services like remote monitoring or smartphone applications, to promote the transition of patients from passive reaction to proactive prevention.

Based on these findings, actionable clinical interventions tailored to HBM dimensions could include: (1) delivering personalized risk communication and recurrence case sharing to enhance perceived threat; (2) providing specific dietary alternatives and short-term achievable goals to reduce perceived barriers; and (3) integrating mHealth tools (e.g., dietary diaries, medication reminders) and professionally guided peer-support networks as external cues and enabling resources to strengthen self-efficacy and support sustained behavior change.

## 5 Conclusion

Based on the HBM, the self-management dilemma of RAP patients manifests as three interrelated dimensions: the perceived threat dimension is characterized by the contradiction between a sense of luck and multiple pressures; the behavioral appraisal dimension shows an imbalance between benefits and barriers; and the behavioral enablers dimension lacks adequate self-efficacy and cues to action. These three dimensions together constitute a structural rupture from “cognition” to “behavior.” Clinical interventions should focus on correcting risk perception, reducing behavioral barriers, enhancing self-efficacy, and establishing proactive cues to action, thereby helping patients translate disease cognition into sustained healthy behaviors.

## 6 Limitations

This study has several limitations. First, the sample was small and recruited from a single region, which may limit generalizability. Second, qualitative analysis is inherently subjective. Third, the use of HBM as the sole framework may have overlooked factors such as social support and psychological status. Fourth, the sample was restricted to RAP patients with prior SAP and ICU admission, limiting generalizability to mild RAP patients without ICU experience. Additionally, some participants did not respond to member checking, possibly due to limited health literacy and educational level. Despite these limitations, the sample was diverse across key demographic and clinical characteristics, supporting the transferability of our findings. Future research should include larger, multi-regional samples and combine quantitative and qualitative methods to further examine cognitive barriers and their influencing factors, as well as to evaluate HBM-based interventions for improving self-management and reducing recurrence rates.

## Data Availability

The raw data supporting the conclusions of this article will be made available by the authors, without undue reservation.
